# A Tale of Two Capacities: Including Children and Decisionally Vulnerable Adults in Biomedical Research

**DOI:** 10.3389/fgene.2019.00289

**Published:** 2019-04-05

**Authors:** Gratien Dalpé, Adrian Thorogood, Bartha Maria Knoppers

**Affiliations:** Centre of Genomics and Policy, McGill University, Montreal, QC, Canada

**Keywords:** capacity, consent, human rights, legally authorized representatives, incompetent adults, data sharing, research, children

## Abstract

The participation of individuals who lack decision-making capacity is essential for advancing genomics research and neuroscience, but raises ethical and legal challenges relating to vulnerability, consent, and exclusion. Capacity differences between populations and individuals, the dynamics of capacity over time, and evolving legal consent and capacity regimes all raise uncertainty for researchers, institutional review boards, and policy makers. We review international ethical and legal best practices for including children and decisionally vulnerable adults in health research. Research ethics norms and literature tend to split such groups into narrow silos, which results in inconsistency and conceptual confusion, or to lump them together, which fails to take into account morally relevant differences. Through a narrative review of international norms, we identify challenges common to both groups, while drawing out distinctions reflecting their opposite capacity trajectories. Our comparison between these two populations clarifies underlying ethical concepts and offers opportunities for critique. Children need protection to foster their long-term autonomy, while decisionally vulnerable adults need to be provided with support in order to exercise their autonomy. This leads to differences in how researchers determine who lacks capacity, who has authority to consent, and what criteria guide such decision-making. We also consider how capacity issues color contemporary research governance debates over broad consent, data protection compliance, data sharing, and the return of individual research results and incidental findings.

## Introduction

Clinical, discovery, and observational research are all essential to improve our understanding of and ability to address disease. The study of neurological and neurodegenerative diseases in particular requires the involvement of participants who lack the capacity to make research-related decisions by themselves. This paper compares ethical and legal safeguards for children and decisionally vulnerable adults in biomedical research. Both groups are ethically and legally considered vulnerable and in need of specific protections against possible violations of their rights and exposure to undue risk. However, these safeguards may too often function to exclude both groups from research participation. Indeed, both groups are traditionally neglected in biomedical research (Canadian Paediatric Society, 2008, p. 707; Jongsma and van de Vathorst, 2015, p. 168; Shepherd, 2016, p. 2; Rahimzadeh et al., 2017, p. 10).

**FIGURE 1:**
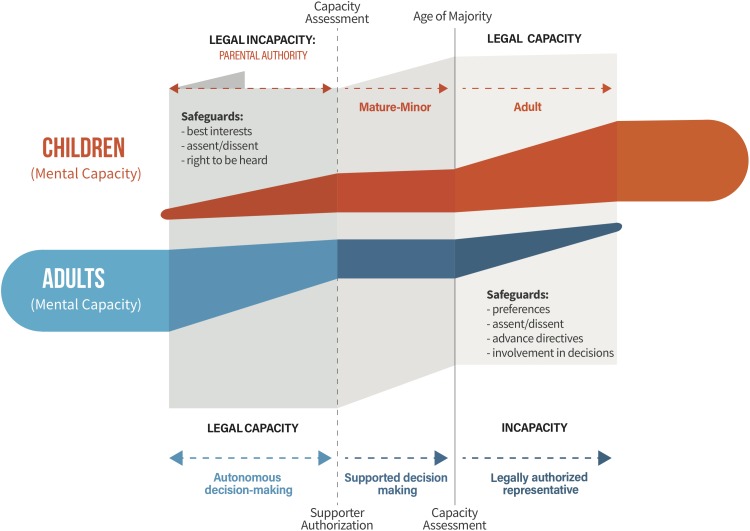
The opposing decision-making capacity trajectories of children and decisionally vulnerable adults in research: ethical and legal considerations. The mental capacity of children normally develops with age. Parents generally have legal authority over their children, who acquire legal capacity at the age of majority. Children may acquire legal capacity at an earlier age for certain types of decisions under mature-minor exceptions. Adults may experience a loss of or fluctuating mental capacity over time. Their legal capacity is generally presumed, until a capacity assessment demonstrates they are no longer able to make certain types of decisions. Under supported decision-making regimes, carers help adults to make their own decisions. For adults who lack legal capacity, LARs make decisions on their behalf. LARs typically have obligations to consult the individual, and to respect certain criteria (e.g., best interests, previous and current wishes, values and beliefs). Note that this Figure constitutes high level generalizations across populations and regulatory frameworks. Refer to Box 1 for definitions.

Despite many similarities, the life and capacity (i.e., decision-making capacity hereafter referred to as capacity) trajectories of these two populations trend in opposite directions, offering a rich opportunity for comparison. The capacity of children generally develops over time, predictably, as part of the natural life course. By contrast, the capacity of adults generally diminishes over time, albeit unpredictably. Loss of capacity may be sudden with stroke or brain injury, steady with neurodegenerative disease, slowly with age, or even not at all. These opposite capacity trajectories are reflected in law. Parents normally have legal authority over their children, who gain authority often at set times in the life course at the legal age of majority. Maturity may, however, be achieved prior to the legally fixed age (e.g., mature minors) (Coleman and Rosoff, 2013). Adults, by contrast, are typically presumed to have legal capacity (United Nations Convention on the Rights of Persons with Disabilities [CRPD], 2006, art. 12(2). A suspected loss of the ability to make decisions triggers a capacity assessment. When it is determined that an individual cannot make a particular decision on their own, consent must be provided by a legally authorized representative (LAR). Figure 1 provides a general summary of ethical and legal safeguards relating to changes in capacity during research.

Research ethics norms and literature tend to split decisionally vulnerable groups into narrow silos, which results in inconsistency and conceptual confusion, or to lump them together, which fails to take into account morally relevant differences. This paper aims to identify challenges common to both children and decisionally vulnerable adults, while drawing out important life course differences.

In the “Human Rights” section, our comparison begins with a review of applicable human rights instruments, which highlight that children need protection to foster their long-term autonomy, while decisionally vulnerable adults with mental disability need to be provided with support in order to exercise their autonomy. The “Protection vs Inclusion” section considers risk and benefit ratios that may restrict the involvement of both populations in research. The “Consent and Capacity in Biomedical Research” section explores a constellation of consent and capacity issues: who has legal authority to consent and the involvement of individuals who lack legal capacity in decision-making (e.g., through assent processes), as well as guidance or restrictions on “proxy” consent for children (normally by parents) and for decisionally vulnerable adults (usually by LARs). It also considers the challenge of defining and assessing functional capacity in biomedical research contexts. The “Capacity Issues in Data-Intensive Research” section considers how capacity colors contemporary research ethics debates in data-intensive research: maintaining “ongoing” consent, data protection, data sharing and access, and the return of individual research results and incidental findings. Each section identifies common protections for both groups, followed by distinct safeguards for children vs. decisionally vulnerable adults, and concludes with a critique where appropriate.

## Methodology

We take an international perspective, to reflect the global nature of biomedical research collaborations. We performed a narrative review of high-level international principles and norms applying to children and decisionally vulnerable adults (Grant and Booth, 2009; Ferrari, 2015). Our aim is not to compare norms between jurisdictions, but rather to compare similarities and distinctions between protections for these two populations. Our narrative review builds on previous comparative analyses of international, regional, and national legislative and policy frameworks that govern informed consent, capacity, data protection, data sharing and access, and the return of results in a biomedical context. Readers interested in more detail about normative convergence and divergence between jurisdictions should refer to these previous publications (Rahimzadeh et al., 2017, 2018; Thorogood et al., 2017, 2018b).

In biomedical contexts, capacity is commonly defined as the ability of the individual to understand relevant information, to appreciate consequences, to reason, and to express a particular decision (Palmer et al., 2017). Legally, by contrast, capacity refers to the *authority* to hold and exercise legal rights and duties (United Nations, 2014, p. 13). We use the terms child and minor interchangeably to mean anyone under the age of legal majority. The United Nations Convention on the Rights of the Child defines child as “a human being below the age of 18 years unless under the law applicable to the child, majority is attained earlier” (United Nations, art. 1). For this paper, we also define decisionally vulnerable adults as persons who have reached the age of majority (e.g., 18 years and older), and who lack the capacity to make research-related decisions by themselves (UC SanDiego, 2019). We prefer a more expansive term that de-emphasizes a misleading binary between capable/incapable/incompetent persons, and highlights that capacity is contextual, depends on a specific decision-making context and support structures, and is dynamic over time. However, we choose to use the terms ‘individual’ or ‘adult’ that ‘lacks capacity’ in the sub-section “Parent/Representative Decision-Making Standards” where we discuss parent/representative decision.

We admit to constructing monolithic conceptual categories of “children” and “decisionally vulnerable adults,” which may ignore the incredible diversity between subgroups and between individuals. While issues of capacity can intersect with other forms of vulnerability (Biros, 2018), laws and research ethics norms tend to recognize these two categories (Jongsma et al., 2015). In terms of developmental stages, biological distinctions are often made between newborns, children, and adolescents. Moreover, children participating in research may suffer from severe neurological disorders permanently curtailing their capacity (Rahimzadeh et al., 2018, p. 477). Adults’ capacity is strongly influenced by cognitive functions. However, no particular cognitive abilities consistently predict the capacity of an adult (Palmer and Savla, 2007, p. 7). Thus, categories of decisionally vulnerable adults are diverse and may include individuals with minor, severe or permanent cognitive impairments (e.g., from birth), and others with fluctuating dementia (e.g., dementia associated with degenerative neurological disorders vs. drug-induced. Drug-induced dementia are distinct as removal of the offending drug generally improves the manifestations).

## Human Rights

Roughly similar international human rights instruments apply to children and decisionally vulnerable adults. While the implementation of these human rights instruments into national legal frameworks varies, they provide a rough indication of general principles and the future direction of international harmonization. The United Nations Treaty Collection (1989), ratified by 196 countries, aims to ensure the full development of children, placing central emphasis on protection and care. The UN CRC defines “best interests” or “well-being” as a guiding principle, where well-being is defined as the enjoyment of the highest attainable standard of health and to facilities for the treatment of illness and rehabilitation of health” (United Nations, art. 3, 12, 24). The United Nations Convention on the Rights of Persons with Disabilities [CRPD] (2006), ratified by 177 countries, applies to all persons with disabilities, including persons with cognitive disabilities. It emphasizes full and effective participation in society, non-discrimination, equal recognition before the law, independence, and the “freedom to make one’s own choices” (United Nations Convention on the Rights of Persons with Disabilities [CRPD], 2006, art. 3). The United Nations (1991), though not binding international law, promote the independence, participation in society, care, self-fulfillment and dignity of the elderly.

The key difference between human rights approaches for children and decisionally vulnerable adults resides in how they address tensions between protection and autonomy. The UN CRC highlights the evolving capacity of the child but also calls for consideration for the extensive rights and obligations of parents as they guide and protect their children (United Nations Treaty Collection (1989), art. 5). Children have a right to be heard, to express their views in all matters concerning them, with due weight given to their age and maturity (United Nations Treaty Collection (1989), art. 12). They have a right to be involved in decisions concerning them, but not the right to make those decisions alone. The CRPD, by contrast, calls for States Parties to provide equal recognition of the legal capacity of persons with disabilities by providing effective support to make their own decisions, and establishing protections that respect disabled adults’ “will and preferences” (United Nations Convention on the Rights of Persons with Disabilities [CRPD], 2006, art. 12; Douglass, 2018, pp. 8–9). Interestingly, the CRPD only mentions “best interests” with regard to children, although this principle is found in many national frameworks applying to decisionally vulnerable adults (United Nations Convention on the Rights of Persons with Disabilities [CRPD], 2006, art. 7.2). The legitimacy (or not) of capacity assessments and substitute decision-making regimes remains controversial (UK government, 2005, p. 4; Johnston and Liddle, 2007; United Nations, 2014; Craigie et al., 2018; Thorogood et al., 2018a). In summary, the focus for children is on protection with increasing participation in decisions as capacity develops. The focus for decisionally vulnerable adults is on supporting their ongoing involvement in decision-making when possible. Another important difference is that the UN CRC considers the family the natural environment for fostering the rights of the child (and so grants deference to parents), whereas the UN CRPD and UN POP are more insistent that the State must directly support and protect the individual human rights of the elderly and disabled.

## Protection vs. Inclusion

The ethical principle of distributive justice calls for making high-quality health care available for all populations, including vulnerable groups. The inclusion of children and decisionally vulnerable adults is often considered necessary in both clinical trials and discovery research to provide evidence-based health care. There are concerns that exclusion of children from research may give rise to a lack of pediatric-specific therapies and impact on the standard of care (Fernandez, 2008; Kim et al., 2017). In order to further develop safe and effective therapies specific to children, there is a need to improve their participation in biomedical research and thus, take into account an appropriate level of protection. Concomitantly, with health care systems encountering aging populations in many Western countries, there is great interest in uncovering the biological basis of neurodegenerative diseases affecting adults such as Alzheimer’s disease and dementia so as to provide a path toward more effective diagnostic tests, preventative measures, and targeted treatments. Advancing research again depends on the participation of both healthy and affected adults in data-intensive studies, from longitudinal population studies of dementia and aging [e.g., The Alzheimer’s Disease Neuroimaging Initiative (ADNI) cohort study], to precision clinical trials. A comprehensive approach to inclusion is reflected by the US National Institutes of Health Inclusion Across the Lifespan Policy that recently came into force, which conditions research funding on appropriate inclusion of younger and older populations in research (NIH, 2017).

Traditional safeguards for vulnerable participants focus on protection from harm and coercion in research. Research ethics principles require a favorable risk-benefit ratio for all research (the risk should be reasonable in relation to anticipated benefits to participants and the importance of the knowledge expected to result) and maintaining minimal or reasonable risk (Cooper and McNair, 2018). Further limitations apply to all research involving decisionally vulnerable participants. In general, such research should involve only minimal risk and burden (International Conference on Harmonization of Technical Requirements for Registration of Pharmaceuticals for Human Use (ICH), 1996, art. 4.8.14b; Council of Europe, 1997, art. 17.ii; World Medical Association [WMA], 2013, art. 28), as additional protections include informed consent from a parent or LAR and respect for assent and dissent (see below). These protections, however, can all too often function to exclude decisionally vulnerable populations from biomedical research. The tension between protection and inclusion is sometimes reframed as an issue of equitable participant selection. For instance, including a vulnerable population is equitable when a high percentage of the patient population with a particular disease also belong to a vulnerable group (e.g., children, adults with cognitive impairment). In contrast, inclusion of participants that lack capacity in a study where the aims are not particularly relevant to this type of vulnerable group could be considered exploitative (Cooper and McNair, 2018).

Decisionally vulnerable adults may face a greater risk of exclusion from research than children. Neglect is generally a greater concern for decisionally vulnerable adults. Children – who are at the beginning of their lives – are more likely to directly benefit from advances in the standard of care. Any improvement will also have a greater actuarial benefit when extrapolated over their life course. Adults of advanced age or disease progression are less likely to directly benefit from research participation, though they may benefit from service improvement when participating in research (Dewing, 2002). Risk-benefit assessments may therefore favor children. One potential solution for decisionally vulnerable adults is to consider personal benefits, such as the enjoyment of sharing their stories, enhanced self-esteem, social activity and sense of making a contribution and being valued (Mills, 1997; Proctor, 2001; Aggarwal et al., 2003; Ross et al., 2005; Edvardsson and Nordvall, 2008).

## Consent and Capacity in Biomedical Research

The ability of individuals to participate in research that may expose them to physical and privacy risks depends on their capacity to provide free and informed consent. Capacity is a precondition of valid informed consent and is typically assessed in relation to a particular decision-making context. Capacity may also fluctuate or diminish over the course of a research project, raising additional legal uncertainty over the ongoing validity of consent. When assessing capacity, there is a need to consider the inherent limitations imposed by the selected capacity assessment tool (e.g., the difficulty of standardizing tailored instruments) (Palmer and Harmell, 2016, 533). Indeed, an important area for future work is to develop, validate, and implement different capacity assessment tools for different high or low risk research contexts (Thorogood et al., 2018b).

### Legal Capacity and the Authority to Consent

Legal capacity refers to the *authority* to hold and exercise legal rights and duties (United Nations, 2014, p. 13). Determining legal capacity for children is relatively straightforward. Parents normally have the legal authority to exercise rights on behalf of children until they reach the age of majority and gain legal capacity. Generally, children cannot consent to participation in research until they have reached the legal age of majority (Knoppers et al., 2016).

We define the mature minor doctrine broadly to include any legal approach that extends legal authority over decisions to children, including medical care and in some cases research participation (e.g., on the basis of age for certain types of decisions, or on the basis of a capacity/maturity test). Following the adoption of the UN CRC in 1989, some countries have modified their legal frameworks to provide more recognition for the autonomy of the child through mature minor exceptions in the form of a legally fixed age for the capacity to provide consent to health care and/or research. For instance, the *Civil Code of Quebec* provides that for the participation of children in research in Quebec (Canada), “[a] minor 14 years of age or over, however, may give consent alone if, in the opinion of the competent research ethics committee, the research involves only minimal risk and the circumstances justify it (Civil Code of Quebec, 2014; art. 21).” The Dutch system uses a dual consent system that allows children of 12 years and older to consent to research in addition with the co-consent of their parents (Knoppers et al., 2016, p. 2; Lepola et al., 2016, p. 2). The *Gillick* case provides that for minors in the United Kingdom, the capacity to give consent to health care is set at 16 years of age (Court of Appeal and Civil Division, 1985). The World Health Organization (CIOMS/WHO) stipulates that the mature minor exceptions may also be extended generally depending on the status of the child, such as those who are pregnant or who live independently (i.e., an emancipated minor) (Council for International Organizations of Medical Sciences (CIOMS) in collaboration with the World Health Organization (CIOMS/WHO), 2016, p. 68) while the UN CRC states that these exceptions are determined by a lowered age threshold for certain types of decisions (United Nations, art. 12).

It is less straightforward to determine when adults lose legal capacity and who is authorized to exercise that capacity in their place. Adults are presumed to have legal capacity unless demonstrated otherwise. Researchers suspecting that an adult participant lacks the capacity to make research related decisions are generally required to carry out a capacity assessment (this presents a number of challenges, discussed in sub-section “Definitions and Assessment of Capacity” below). Where the assessment establishes that the adult participant is unable to make research-related decisions, a LAR can make decisions on their behalf. LARs may be authorized by an advance directive, court order, or substitute decision-making statute (International Conference on Harmonization of Technical Requirements for Registration of Pharmaceuticals for Human Use (ICH), 1996, art. 4.8.5; Council of Europe, 1997, art. 6.3; UNESCO, 2003, art. 8(b); World Medical Association [WMA], 2013, art. 28; Council for International Organizations of Medical Sciences (CIOMS) in collaboration with the World Health Organization (CIOMS/WHO), 2016, 61, guideline 16). Laws are not always clear about who (if anyone) can act as a LAR for biomedical research (Thorogood et al., 2018b). Even where laws are clear, it can be practically difficult to identify a LAR. Moreover, the idea that LARs make decisions *on behalf* of decisionally vulnerable adults, like parents make decisions on behalf of children, is controversial in light of the CRPD General comment No. 1 (United Nations, 2014), which criticizes proxy consent processes and instead promotes “supported decision-making” for all persons with mental disability to enable them to exercise and maintain their legal capacity, and realize their own choices, thus providing legal recognition of the spectrum of capacity. The *Nuffield Council on Bioethics*, the *UK Mental Capacity Act* and the UN CRPD highlight the importance of exploring the possibilities for supporting individual decisions before a person is declared incompetent (UK government, 2005; United Nations Convention on the Rights of Persons with Disabilities [CRPD], 2006, art. 12(3); Nuffield Council on Bioethics, 2009; United Nations Treaty Collection, 1989, p. 2). In conclusion, dedicated resources and expertise are often needed to involve decisionally vulnerable adults in research.

### Shared Decision-Making: Assent/Dissent

Research obligations to seek assent and to respect dissent are similar in principle for both children and decisionally vulnerable adults. International and national research ethics guidelines also encourage researchers to establish the individual’s wish to participate (assent) even though consent is provided by a parent or LAR (International Conference on Harmonization of Technical Requirements for Registration of Pharmaceuticals for Human Use (ICH), 1996, art. 4.8.12; World Medical Association [WMA], 2013, art. 29; Canada, Tri-Council Policy Statement [TCPS], 2014, art. 3.10; Council for International Organizations of Medical Sciences (CIOMS) in collaboration with the World Health Organization (CIOMS/WHO), 2016, 61, guideline 16). Researchers should also respect dissent, which may appear as verbal or physical signs of distress. The Canadian research ethics guidelines defines distress as signs of anxiety, depression, embarrassment, or acute stress reaction (Tri-Council Policy Statement [TCPS], 2014, art. 3.10; See also International Conference on Harmonization of Technical Requirements for Registration of Pharmaceuticals for Human Use (ICH), 1996, art. 4.8.14; Draucker et al., 2009; World Medical Association [WMA], 2013, art. 29). Assent/dissent processes address to some extent the child’s right to be heard, and the decisionally vulnerable adult’s right to be supported in (or at least involved in) decision-making (Herring, 2013, 156–160; Mäki-Petäjä-Leinonen, 2016).

Assent in pediatric research is viewed as a means of developing the engagement of children together with their parents in research, but without denying parents their authority over certain decisions (Wilfond and Diekema, 2012; Giesbertz et al., 2014). Assent becomes more important for children as they mature (International Conference on Harmonization of Technical Requirements for Registration of Pharmaceuticals for Human Use (ICH), 1996, art. 4.8.12; Council of Europe, 1997, art. 6.2; UNESCO, 2003, art. 8(c); World Medical Association [WMA], 2013, art. 29; Tri-Council Policy Statement [TCPS], 2014, art. 3.3; Council for International Organizations of Medical Sciences (CIOMS) in collaboration with the World Health Organization (CIOMS/WHO), 2016, guideline 17). The increasing maturity of adolescents near the age of majority can even mean assent is an ethical ‘co-consent’ requirement (Tri-Council Policy Statement [TCPS], 2014, p. 32). In biomedical research contexts, consent processes should ensure that researchers, family members, and carers all provide support to persons with dementia to assist participation in decision-making (Council of Europe, 1999; United Nations Convention on the Rights of Persons with Disabilities [CRPD], 2006; United Nations, 2014; Murphy et al., 2015). LARs are generally required by law to respect and consider the instructions and wishes expressed by the adult while still capable. They also generally have procedural duties to consult the individual and take their current wishes into account if they can be expressed, as well as duties to seek assent and respect dissent (See Box 1) (Thorogood et al., 2018b, p. 1338).

**Box 1. Key definitions.**

• **Legal capacity:** authority to hold and exercise legal rights and duties.

• **Mature minor exception:** legal regimes that authorize minors below the age of majority to make certain types of decisions (e.g., health-care decisions), usually dependent on their age, maturity and/or capacity.

• **Supported decision-making:** legal regimes where carers provide support for adults to enable them to exercise their legal capacity.

• **LAR:** legally authorized representative. An individual or body authorized by law to make decisions on behalf of an individual who lacks legal capacity.

• **Mental capacity:** in biomedical contexts, a person’s ability to understand relevant information, to appreciate consequences, to reason, and to express a decision.

• **Capacity assessment:** assessment of an individual’s functional capacity to make a specific decision (e.g., to consent to research participation).

• **Advanced directives:** instructions, subject to certain formalities, made in advance of a loss of capacity that direct or guide LAR decision-making.

• **Assent:** an expression of willingness to participate (e.g., to participate in research).

• **Dissent:** an expression of opposition to participate (e.g., to participate in research).

Practically, the involvement of family members or carers can facilitate assent processes. Assent can also be supported by developing communication tools tailored to age, condition, and levels of capacity (e.g., simplified forms, visual memory aids, interactive or educational approaches, or re-explaining misunderstood information (Tait et al., 2007; Diener et al., 2013; Nishimura et al., 2013; Sweet et al., 2014; Simpson, 2015; Prusaczyk et al., 2017)). Other factors might also be taken into account, such as individual circumstances, emotional and psychological maturity, and support situation (Council for International Organizations of Medical Sciences (CIOMS) in collaboration with the World Health Organization (CIOMS/WHO), 2016, p. 67). There is also uncertainty over dissent. It remains unclear if researchers should stop participation at the first sign of distress or only where distress is serious and sustained. Given the complexity involved in designing and administering assent and dissent, there is woefully little guidance on these topics for investigators and research ethics committees (Civil Code of Quebec, 2014, art. 21; See also Wendler, 2006).

### Parent/Representative Decision-Making Standards

The best interests is a guiding principle for representatives of individuals who lack legal capacity to make decisions on their own. For children, this is the primary criterion for courts, health care providers, and parents, though the latter are typically extended substantial discretion to decide what is best for the child. A child’s best interests may be broken down into a hierarchy including individual medical benefit, familial medical benefit (which could indirectly benefit the child), and benefitting other pediatric patients (Rahimzadeh et al., 2018, p. 477). The most difficult aspect for parents is to balance the best interests and evolving capacity of children to make their own decisions. In contrast, LARs making decisions on behalf of an adult who lacks legal capacity are often legally required to first consider the person’s past and current wishes, beliefs, values and well-being. It is usually only in the absence of such preferences that the best interests (i.e., welfare) of the person considered (Archibald and Lemmens, 2008, pp. 152–159). They also provide an alternative source of guidance for LARs that can be most relevant when traditional considerations of best interests are unclear. In practice, however, consideration of wishes, beliefs and values may not always be particularly helpful in research contexts, as they are often unknown for this specific context. Moreover, the best interests test is not always a helpful guide, especially for non-therapeutic research aiming to create generalizable knowledge, even if the risks are low (Berger, 2011, p. 46). The application of the best interests may be more problematic for the elderly unable to make research decisions than for children, as such persons with advanced age and disease are generally less likely to benefit from eventual or future advances in non-therapeutic research and health care. Thus, parents are primarily expected to focus on best interests while this consideration becomes subsidiary to wishes, beliefs, values and well-being for decisionally vulnerable adults.

One consent consideration that primarily applies to adults who lack capacity is advance planning. In many contexts, adults may specify their wishes or instructions (e.g., for care) in advance of a loss of capacity. Some jurisdictions have laws that allow individuals to articulate instructions in a legally binding “advance directive” that must be respected by third party decision makers (e.g., health care providers) and by LARs. However, the scope of such laws is often limited to certain types of decisions (e.g., health care treatment), and their application in research contexts can be uncertain. Even in the absence of advance directives, past wishes are still typically an important legally binding consideration for LARs. While past wishes are not addressed in many research norms, some suggest that researchers (and not just LARs) should take into account the already known wishes of adults who have lost the capacity to consent (Tri-Council Policy Statement [TCPS], 2014, art. 3.9). Depending on the jurisdiction, formally expressed instructions (e.g., written in an advance directive), may or may not have priority over informally expressed wishes. Advance planning may also be relevant in limited circumstances for children, e.g., older, chronically ill children in pediatric-palliative care contexts (Liberman et al., 2014).

In short, while parents have broad discretion to decide what is best for their child, LARs for adults who lack capacity have to follow a more structured decision-making process than parents and are increasingly expected to act as agents, who carry out the will and preferences of the person.

### Definitions and Assessment of Capacity

Researchers have an obligation to ensure that individuals consenting to research participation have the capacity to do so. In cases where it is determined that the individual does not have the capacity to consent to research, researchers will need to seek consent from a parent or LAR. Determining if an individual has the capacity to make a decision in the context of biomedical research differs between children and decisionally vulnerable adults.

The law may also provide the possibility for courts or physicians to assess the maturity of a child on a case-by-case basis. The province of Ontario (Canada) provides that a “person is capable with respect to a treatment, admission to a care facility or a personal assistance service if the person is able to understand the information that is relevant to making a decision about the treatment, admission or personal assistance service, as the case may be, and able to appreciate the reasonably foreseeable consequences of a decision or lack of decision (Ontario Health Care Consent Act, 2017)”. Some jurisdictions set a lower legally fixed age to consent to medical care, which is also commonly used in research contexts. This makes it more straightforward to determine if legal authority rests with a parent or with a child but can seem arbitrary as children mature at different rates. Risks to the child’s autonomy, however, are mitigated by assent and dissent processes, discussed above, which ensure children are part of decision-making even if they do not have the sole legal authority to consent.

Clinicians and researchers who suspect an adult is unable to consent to treatment or research participation must carry out a capacity assessment, which adduces evidence that the adult lacks the functional capacity to consent. Functional capacity is the person’s ability to understand relevant information, to appreciate consequences, to reason, and to express a decision (Palmer et al., 2017). If the adult does not have the capacity to provide consent, researchers are required to identify and obtain consent from a LAR. Like the decision to determine “maturity” for minors, a common challenge for researchers in the adult context is knowing when and how to assess capacity (Warner and Nomani, 2008). Laws, research policies and study protocols are often silent about when and how to assess a lack of capacity (Biros, 2018).

## Capacity Issues in Data-Intensive Research

The data-intensive nature of modern genetic and health research has raised a number of legal and ethical issues ranging from consent given by participants, privacy of data, data sharing research and the return of individual results. These issues may present practical challenges for vulnerable populations. While “broad consent” – i.e., consent where the research uses of samples/data cannot be fully specified at the time of consent, accompanied by ongoing oversight – has gained wide acceptance, debate continues over the appropriate standard for informed consent. Rich bioinformatics data tend to be unique to an individual and potentially identifying, raising challenges for privacy protection. Uncertainty also persists over if, when, and how researchers should return individual findings of health relevance to participants (Burke et al., 2018). Finally, researchers are increasingly expected to share their individual-level research data broadly – to ensure data-intensive research is rigorous, reproducible, and efficient (Knoppers and Thorogood, 2017; Taichman et al., 2017). Data sharing has given new dimensions to consent and privacy concerns, prompting advances in network technologies and security, access, and research “governance” practices to manage these risks (GA4GH, 2014). Data-intensive biomedical research involving individuals with limited decisional capacity raises additional legal and ethical concerns over their autonomy, protection and inclusion (Gehlert and Mozersky, 2018).

### “Ongoing” Consent

It is a basic principle that consent in biomedical research is ongoing and must be maintained throughout a study. Participants are generally free to withdraw their consent at any time. For discovery or observational research involving data and samples, this principle is theoretically strained where there is no ongoing interaction with the participant. There are also practical challenges and limitations to withdrawal of samples and data. The opposite capacity trajectories of children and adults raise additional challenges for longitudinal research.

Where a parent consented to research on behalf of a child, who then attains legal majority during the course of a study, the traditional rule is that researchers should renew consent from the individual (Tri-Council Policy Statement [TCPS], 2014, p. 32; Council for International Organizations of Medical Sciences (CIOMS) in collaboration with the World Health Organization (CIOMS/WHO), 2016, p. 67, guideline 17). Such a rule presents challenges for discovery and observational studies, especially those without continuous engagement with participants. Practical difficulties of re-contacting new adults or engaging them about the importance of continued participation can drastically undermine retention rates if re-consent is required (Knoppers et al., 2016). Notification with the opportunity to opt-out has therefore been proposed as an alternative to re-consent upon attaining the majority (Hens et al., 2013).

Adults may lose capacity during the course of a study. In experimental research, consent must be renewed by the LAR (World Medical Association [WMA], 2013, art. 30; Council for International Organizations of Medical Sciences (CIOMS) in collaboration with the World Health Organization (CIOMS/WHO), 2016, guideline 16; Health Canada, 2017, art. 4.8.2). Often the individual will not practically be able to continue participating without the practical support of a representative or carer. In discovery or observational research, however, researchers will not always be aware the individual has lost capacity (Canadian Longitudinal Study on Aging [CLSA], 2008, p. 52). Even if there is regular interaction (e.g., data collection or health assessments), it is not always clear when and how to assess capacity. The question therefore arises: does consent to use samples and data endure past the loss of capacity? Arguably, such consent should continue to be respected as a written expression of the individual’s wishes (Council for International Organizations of Medical Sciences (CIOMS) in collaboration with the World Health Organization (CIOMS/WHO), 2016; Thorogood et al., 2018a). This leads to additional questions: should the enduring nature of consent be made explicit at the time of consent? Should family members or LARs be allowed to override these wishes (Genomics England, 2017, p. 80, p. 83; Rahimzadeh et al., 2018, p. 477; Thorogood et al., 2018b, p. 1339)? On the one hand, the authority of the representative to exercise the individual’s rights should be respected; on the other hand, LAR withdrawal would override the individual’s previously expressed wishes.

### Data Protection

Data-intensive research increasingly attracts the application of data protection laws. These laws generally require consent to the collection, use and disclosure of personal data, and that personal data be kept confidential and secure. Because they depend on consent, data protection frameworks must apply differently when it comes to safeguarding the rights and interests of children and decisionally vulnerable adults. The 2018 EU *General Data Protection Regulation* (GDPR), an influential data protection law, uses an age-based approach in which consent from minors of 16 and up is required for processing personal data (European Parliament and Council, 2018, art. 4, 8). The GDPR sets the age of consent for data processing at 16 years and more (European Parliament and Council, 2018, art. 8). Member states can deviate to as low as 13 years (Lievens and Milkaité, 2017; European Parliament and Council, 2018, art. 8). The GDPR provides that processing special categories of personal data from incapable adults (e.g., sensitive data such as genetic data) shall not be authorized unless the processing is necessary to “protect the vital interests of the data subject” or “when data processing occurs in the context of scientific research.” The GDPR defines sensitive data as “data revealing racial or ethnic origin, political opinions, religious or philosophical beliefs, or trade union membership, and the processing of genetic data, biometric data for the purpose of uniquely identifying a natural person, data concerning health or data concerning a natural person’s sex life or sexual orientation (European Parliament and Council, 2018, art. 9.2(c)(j))”. Data protection laws tend to enable a LAR to give or withdraw consent on behalf of an incapable adult to participate in data-centric research (Archibald and Lemmens, 2008, p. 162). For minors, the processing of personal data does not necessarily need the consent of parents but is subject to specific protections (European Parliament and Council, 2018, Recital 38).

### Data Sharing

Data sharing is the (sometimes broad or public) exchange of individual-level genomic and health-related data generated as part of research or clinical testing. Genomic and biomedical researchers are encouraged or required to share by funders, journals and institutions to foster collaboration, improve the rigor and reproducibility of research, and reduce duplicative effort (Taichman et al., 2017; National Academies of Sciences, Engineering, and Medicine [NASEM], 2018a). Clinical institutions, too, are developing innovative ways to share patient data to facilitate diagnosis or to support research, and are being encouraged to do so by professional societies (ACMG, 2017). Privacy safeguards (coding and anonymization) and security safeguards (locks, firewalls, data access processes, and data access agreements) can limit informational risks for data subjects (Council for International Organizations of Medical Sciences (CIOMS) in collaboration with the World Health Organization (CIOMS/WHO), 2016, guideline 12; International Council for Harmonization of Technical Requirements for Pharmaceuticals for Human Use (ICH), 2017, 18). Research ethics and privacy regulations may also require consent for sharing rich individual-level data (UNESCO, 2003, art. 8).

One might argue that restrictions or additional safeguards should be imposed when sharing data from decisionally vulnerable populations. Indeed, special privacy and security safeguards are required for the personal data of children under the EU GDPR, but are not defined (European Parliament and Council, 2018, art. 6.1(f)). These protections may be justified by the increased likelihood of new privacy risks emerging over a child’s lifetime. Alternatively, principles of inclusion suggest that researchers and clinicians should give these populations equal if not greater opportunities to participate in transparent and reproducible research (Rahimzadeh et al., 2018; Thorogood et al., 2018b). Indeed, the scientific value of data is greater when it concerns hard to study populations. Data sharing can also help to avoid duplicative studies exposing vulnerable participants to unnecessary physical risks. There are already numerous examples of efforts to share research or clinical data from pediatric populations (Children Oncolgy Group [COG], 2018; Pediatric Trials Network Data Sharing, 2018), as well as populations of adults with neurodegenerative disease (Canadian Longitudinal Study on Aging [CLSA], 2017; Alzheimer’s Disease Neuroimaging Initiative [ADNI], 2018; Canadian Open Neuroscience Platform [CONP], 2018).

Consent is often generally required by research and privacy regulations to share identifiable data. Data sharing involving decisionally vulnerable individuals must therefore contend with challenges relating to the capacity. They depend on others to protect their privacy and, where possible, to protect their informational autonomy.

One can imagine data sharing safeguards analogous to research safeguards. Parents or LARs can be asked to consent to data sharing on behalf of the individual. Children and represented adults can be involved in the decision (Rahimzadeh et al., 2018, p. 476; Thorogood et al., 2018b, p. 1338). Adults should “as far as possible take part in the authorization procedure,” and a child’s opinion “should be taken into consideration as an increasingly determining factor in proportion to age and degree of maturity” (UNESCO, 2003, art. 8). Moreover, the ICGC encourages assent in their access policy (International Cancer Genome Consortium [ICGC], 2012).

Overall, however, it remains unclear when and how to involve decisionally vulnerable individuals in research and data sharing decisions (Wendler, 2006; Rahimzadeh et al., 2017). Data sharing decisions are often embedded in broader decisions to seek research and testing, and are comparatively low risk and high complexity. Is assent feasible for these types of decisions? Should children be re-consented for data sharing once they reach the age of majority? Should researchers allow LARs to withdraw data where a decisionally vulnerable adult previously expressed a preference for sharing through consent?

### Return of Individual Research Results and Incidental Findings

The ethical and legal obligations of researchers to return information of health relevance to participants have received significant attention of late, particularly in genomics research (National Academies of Sciences, Engineering, and Medicine [NASEM], 2018b). The basic ethical principle is that researchers should return analytically valid, clinically valid, and actionable information to participants (Knoppers et al., 2015, p. 556). Distinctions are sometimes drawn between individual results related to the primary research question, incidental findings unrelated to the research question, or secondary findings that are unrelated to but are actively analyzed (Knoppers et al., 2015, p. 554). Return in diverse research contexts is complicated by resource limitations, uncertainty over individual preferences to know (or not), and determining what meets the criteria (Knoppers et al., 2015, p. 556). Often, researchers are required to develop an ethics-approved plan to return (or not) individual findings, and to explain this plan during informed consent (UNESCO, 2003, art. 6; United Nations Economic and Social Council, 2004, art. 6; World Medical Association [WMA], 2013, art. 32 (implied); Tri-Council Policy Statement [TCPS], 2014, art. 13.2; Council for International Organizations of Medical Sciences (CIOMS) in collaboration with the World Health Organization (CIOMS/WHO), 2016, guideline 11; International Council for Harmonization of Technical Requirements for Pharmaceuticals for Human Use (ICH), 2017, 18, art. 5–6).

For decisionally vulnerable participants, questions arise over the discretion of parents or LARs to consent to or refuse return of results (Wolf et al., 2018). As a general principle, researchers should grant representatives full discretion to exercise the rights of the individual, recognizing that representatives have an ethical and legal duty to act in the individual’s interests (UNESCO, 2000, art. 51; Boycott et al., 2015). The familial nature of genetic risk, however, can raise conflicts of interest between the individual’s health and a biologically related representative’s informational autonomy. For children, some professional associations hold that parents should not be allowed to refuse actionable results for serious conditions that occur during childhood (ACMG, 2015; Botkin et al., 2015 (for genes of clinical interest); van El et al., 2013; Knoppers et al., 2014; Boycott et al., 2015). To fail to do so could be considered medical neglect under child protection legislation. This logic can be extended to results with implications for the parents’ health or reproductive choices, which may indirectly impact the well-being of the child (Rigter et al., 2013). Returning results about adult onset conditions to children raises an additional tension between the child’s future health and their “right” to an open future. Some policies recommend waiting until the child reaches adulthood before offering such results (Knoppers et al., 2014; Zawati et al., 2014). A concern with this approach is that the child may never receive the information. For mature minors, researchers also need to consider if results should be returned directly to the parents and/or the minor.

Return of results for decisionally vulnerable adults should take in consideration not only their best interests but also their previously expressed wishes, values and beliefs (Thorogood et al., 2018b). Tensions can arise for researchers or LARs if the individual previously expressed a wish not to share familial risk information with family members (Boycott et al., 2015, recommendation 3.3). Wolf et al. recommend that researchers and LARs respect the participant’s expressed preferences about sharing his or her data with family members, unless there is an overriding health interest for the family member (Wolf et al., 2018, 92–94).

## Conclusion

As Charles Dickens observed, the noisiest authorities of any era will insist it be received in the “superlative degree of comparison only” (Dickens, 1998). This is true for the hype surrounding both the promise and the risks of modern health research (Caulfield et al., 2013). With the aim of providing some clarity and calm, we have identified common legal and policy considerations for the participation of decisionally vulnerable participants in biomedical research. A central human rights consideration for both children and decisionally vulnerable adults is inclusion (see “Human Rights” section), in addition to protection and respect for persons. The involvement of these groups in research is necessary to ensure they too benefit from the progress of science and improvements in care (Canadian Paediatric Society, 2008; Shepherd, 2016; Thorogood et al., 2019). Our comparison identifies morally relevant similarities and distinctions between decisionally vulnerable populations that can inform the design of research regulation and governance. For adults “who have previously lived more autonomous lives,” safeguards should be sensitive to life-course considerations and biographical context (Jongsma and Schweda, 2018). For children, it is important to avoid implying that normal states of development are pathological, or alternatively that mental capacity is synonymous with maturity (Jongsma and Schweda, 2018, 417). While it is tempting to think of capacity as exercised at a particular moment in time, Jeremy Waldron has convincingly argued that giving equal value to each human life requires us to consider life as a whole, across a developmental trajectory (Waldron, 2017, 233). Another problem to avoid is conflating decisional vulnerability with broader vulnerability considerations (taking a narrow neurocognitive view), which fails to take into account morally relevant differences. The solution is for research governance to leave room for sensitivity to diverse physical, relational, and cognitive dimensions of vulnerability.

## Author Contributions

GD and AT contributed equally to the article. All authors made a substantial contribution to the analysis and interpretation of the data, provided a critical revision and agreed with the final version of the manuscript and all aspects of the work.

## Conflict of Interest Statement

The authors declare that the research was conducted in the absence of any commercial or financial relationships that could be construed as a potential conflict of interest.

## References

[B1] ACMG (2015). ACMG policy statement: updated recommendations regarding analysis and reporting of secondary findings in clinical genome-scale sequencing. *Genet. Med.* 17 68–69. 10.1038/gim.2014.151 25356965

[B2] ACMG (2017). Laboratory and clinical genomic data sharing is crucial to improving genetic health care: a position statement of the American College of Medical Genetics and Genomics. *Genet. Med.* 19 721–722. 10.1038/gim.2016.196 28055021

[B3] Aggarwal N. Vass A. A. Minardi H. A. Ward R. Garfield C. Cybyk B. (2003). People with dementia and their relatives: personal experiences of Alzheimer’s and of the provision of care. *J. Psychiatr. Ment. Health Nurs.* 10 187–197. 10.1046/j.1365-2850.2003.00550.x 12662335

[B4] Alzheimer’s Disease Neuroimaging Initiative [ADNI] (2018). *Alzheimer’s Disease Neuroimaging Initiative.* Available at: http://adni.loni.usc.edu/ (accessed October 25, 2018).

[B5] Archibald T. Lemmens T. (2008). Data collection from legally incompetent subjects: a paradigm legal and ethical challenge for population databanks. *Health Law J.* 36.

[B6] Berger J. T. (2011). Is best interests a relevant decision making standard for enrolling non-capacitated subjects into clinical research? *J. Med. Ethics* 37 45–49. 10.1136/jme.2010.037515 20952491

[B7] Biros M. (2018). Capacity, vulnerability, and informed consent for research. *J. Law Med. Ethics* 46 72–78. 10.1177/1073110518766021 29991882 PMC6035898

[B8] Botkin J. R. Belmont J. W. Berg J. S. Berkman B. E. Bombard Y. Holm I. A. (2015). Points to consider: ethical, legal, and psychosocial implications of genetic testing in children and adolescents. *Am. J. Hum. Genet.* 97 6–21. 10.1016/j.ajhg.2015.05.022 26140447 PMC4570999

[B9] Boycott K. Hartley T. Adam S. Bernier F. Chong K. Fernandez B. A. (2015). The clinical application of genome-wide sequencing for monogenic diseases in Canada: Position Statement of the Canadian College of Medical Geneticists. *J. Med. Genet.* 52 431–437. 10.1136/jmedgenet-2015-103144 25951830 PMC4501167

[B10] Burke W. Beskow L. M. Trinidad S. B. Fullerton S. M. Brelsford K. (2018). Informed consent in translational genomics: insufficient without trustworthy governance. *J. Law Med. Ethics* 46 79–86. 10.1177/1073110518766023 29962827 PMC6023399

[B11] Canadian Longitudinal Study on Aging [CLSA] (2008). *CLSA Protocol – Full Study Design, and Baseline.* Available at: https://clsa-elcv.ca/doc/511 (accessed September 7, 2017).

[B12] Canadian Longitudinal Study on Aging [CLSA] (2017). *Canadian Longitudinal Study on Aging.* Available at: https://www.clsa-elcv.ca/ (accessed September 7, 2017).

[B13] Canadian Open Neuroscience Platform [CONP] (2018). *The Neuro Joins Neuroscience Data Sharing Partnership.* Available at: https://www.mcgill.ca/neuro/channels/news/neuro-joins-neuroscience-data-sharing-partnership-285125 (accessed May 9, 2018).

[B14] Canadian Paediatric Society (2008). Ethical issues in health research in children?: position statement. *Paediatr. Child Health* 13 707–712. 10.1093/pch/13.8.707 19436527 PMC2606083

[B15] Caulfield T. Chandrasekharan S. Joly Y. Cook-Deegan R. (2013). Harm, hype and evidence: ELSI research and policy guidance. *Genome Med.* 5:21. 10.1186/gm425 23534337 PMC3707044

[B16] Children Oncolgy Group [COG] (2018). *COG Data Sharing.* Available at: https://www.childrensoncologygroup.org/index.php/data-sharing (accessed November 8, 2018).

[B17] Civil Code of Quebec (2014). Available at: https://www.canlii.org/en/qc/laws/stat/lrq-c-c-1991/latest/lrq-c-c-1991.html#sec1370 (accessed June 18, 2014).

[B18] Coleman D. L. Rosoff P. M. (2013). The legal authority of mature minors to consent to general medical treatment. *Pediatrics* 131 786–793. 10.1542/peds.2012-2470 23530175

[B19] Cooper J. A. McNair L. (2018). A practical approach to including adults unable to consent in research. *J. Empir. Res. Hum. Res. Ethics* 13 185–186. 10.1177/1556264618761693 29448868

[B20] Council for International Organizations of Medical Sciences (CIOMS) in collaboration with the World Health Organization (CIOMS/WHO) (2016). *International Ethical Guidelines for Health-Related Research Involving Humans.* Available at: https://cioms.ch/wp-content/uploads/2017/01/WEB-CIOMS-EthicalGuidelines.pdf (accessed March 1, 2017). 40523065

[B21] Council of Europe (1997). *Convention for the Protection of Human Rights and Dignity of the Human Being with Regard to the Application of Biology and Medicine.* Available at: https://www.coe.int/en/web/conventions/full-list/-/conventions/treaty/164 (accessed August 23, 2013).

[B22] Council of Europe (1999). *Recommendation No. R (99) 4 on Principles Concerning the Legal Protection of Incapable Adults.* Available at: https://search.coe.int/cm/Pages/result_details.aspx?ObjectID=09000016805e303c (accessed March 22, 2017).

[B23] Court of Appeal and Civil Division (1985). Gillick v. West Norfolk and Wisbech area health authority. *All Engl. Law Rep.* 1 533–559. 11648530

[B24] Craigie J. Bach M. Gurbai S. Kanter A. Kim S. Y. H. Lewis O. (2018). Legal capacity, mental capacity and supported decision-making: report from a panel event. *Int. J. Law Psychiatry* 62 160–168. 10.1016/j.ijlp.2018.09.006 30389184 PMC6372113

[B25] Dewing J. (2002). From ritual to relationship: a person-centred approach to consent in qualitative research with older people who have a dementia. *Dementia* 1 157–171. 10.1177/147130120200100204

[B26] Dickens C. (1998). *A Tale of Two Cities: Unabridged Edition.* Mineola, NY: Dover Publications.

[B27] DienerL.Hugonot-DienerL.AlvinoS.BaeyensJ. P.BoneM. P.ChiritaD. (2013). Guidance synthesis. Medical research for and with older people in Europe: proposed ethical guidance for good clinical practice: ethical considerations. *J. Nutr. Health Aging* 17 625–627. 10.1007/s12603-013-0340-0 23933874

[B28] Douglass A. (2018). Rethinking necessity and best interests in New Zealand mental capacity law. *Med. Law Int.* 18 3–34. 10.1177/0968533218762240

[B29] Draucker C. B. Martsolf D. S. Poole C. (2009). Developing distress protocols for research on sensitive topics. *Arch. Psychiatr. Nurs.* 23 343–350. 10.1016/j.apnu.2008.10.008 19766925

[B30] Edvardsson D. Nordvall K. (2008). Lost in the present but confident of the past: experiences of being in a psycho-geriatric unit as narrated by persons with dementia. *J. Clin. Nurs.* 17 491–498. 10.1111/j.1365-2702.2006.01826.x 18205681

[B31] European Parliament and Council (2018). *Regulation on the Protection of Natural Persons with Regard to the Processing of Personal Data and on the Free Movement of Such Data, and REPEALING DIRECTIVE 95/46/EC (General Data Protection Regulation).* Available at: http://eur-lex.europa.eu/legal-content/EN/TXT/HTML/?uri=CELEX:32016R0679&from=FR (accessed October 12, 2017).

[B32] Fernandez C. (2008). Ethical issues in health research in children. *Paediatr. Child Health* 13 707–712. 10.1093/pch/13.8.707 19436527 PMC2606083

[B33] Ferrari R. (2015). Writing narrative style literature reviews. *Med. Writ.* 24 230–235. 10.1179/2047480615Z.000000000329

[B34] GA4GH (2014). *Framework for Responsible Sharing of Genomic and Health-Related Data. Global Alliance for Genomics and Health (GA4GH)*. Available at: https://ga4gh.edit.sanger.ac.uk/genomic-data-toolkit/regulatory-ethics-toolkit/framework-forresponsible-sharing-of-genomic-and-healthrelated-data/ (accessed May 11, 2017).

[B35] Gehlert S. Mozersky J. (2018). Seeing beyond the margins: challenges to informed inclusion of vulnerable populations in research. *J. Law Med. Ethics* 46 30–43. 10.1177/1073110518766006 30093794 PMC6077979

[B36] Genomics England (2017). *The 100,000 Genomes Project Protocol. The 100,000 Genomes Project.* Available at: https://www.genomicsengland.co.uk/100000-genomes-project-protocol/ (accessed November 2, 2017).

[B37] Giesbertz N. A. A. Bredenoord A. L. van Delden J. J. M. (2014). Clarifying assent in pediatric research. *Eur. J. Hum. Genet.* 22 266–269. 10.1038/ejhg.2013.119 23756442 PMC3895639

[B38] Grant M. J. Booth A. (2009). A typology of reviews: an analysis of 14 review types and associated methodologies: a typology of reviews. *Maria J. Grant Andrew Booth. Health Inform. Librar. J.* 26 91–108. 10.1111/j.1471-1842.2009.00848.x 19490148

[B39] Health Canada (2017). *Good Clinical Practice: Integrated Addendum to E6(R1), ICH Topic E6(R2).* Available at: http://www.hc-sc.gc.ca/dhp-mps/prodpharma/applic-demande/guide-ld/ich/efficac/e6r2-step4-eng.php (accessed October 5, 2017).

[B40] Hens K. Van El C. E. Borry P. Cambon-Thomsen A. Cornel M. C. Forzano F. (2013). Developing a policy for paediatric biobanks: principles for good practice. *Eur. J. Hum. Genet.* 21 2–7. 10.1038/ejhg.2012.99 22713814 PMC3533257

[B41] Herring J. (2013). *Caring and the Law.* Oxford: Hart Publishing.

[B42] International Cancer Genome Consortium [ICGC] (2012). *E.1 Informed Consent, Access and Ethical Oversight.* Available at: http://icgc.org/icgc/goals-structure-policies-guidelines/e1-informed-consent-access-and-ethical-oversight (accessed March 22, 2018).

[B43] International Conference on Harmonization of Technical Requirements for Registration of Pharmaceuticals for Human Use (ICH) (1996). *Guideline for Good Clinical Practice E6(R1).* Available at: https://www.ich.org/fileadmin/Public_Web_Site/ICH_Products/Guidelines/Efficacy/E6/E6_R1_Guideline.pdf (accessed August 10, 2017).

[B44] International Council for Harmonization of Technical Requirements for Pharmaceuticals for Human Use (ICH) (2017). *Guideline on Genomic Sampling and Management of Genomic Data – E18.* Available at: http://www.ich.org/fileadmin/Public_Web_Site/ICH_Products/Guidelines/Efficacy/E18/E18EWG_Step4_Guideline_2017_0803.pdf (accessed October 5, 2017).

[B45] Johnston C. Liddle J. (2007). The mental capacity act 2005: a new framework for healthcare decision making. *J. Med. Ethics* 33 94–97. 10.1136/jme.2006.016972 17264196 PMC2598235

[B46] Jongsma K. Bos W. van de Vathorst S. (2015). Morally relevant similarities and differences between children and dementia patients as research subjects: representation in legal documents and ethical guidelines. *Bioethics* 29 662–670. 10.1111/bioe.12195 26481208

[B47] Jongsma K. Schweda M. (2018). Return to childhood? Against the infantilization of people with dementia. *Bioethics* 32 414–420. 10.1111/bioe.12458 30106171

[B48] Jongsma K. R. van de Vathorst S. (2015). Beyond competence: advance directives in dementia research. *Monash Bioeth. Rev.* 33 167–180. 10.1007/s40592-015-0034-y 26458366 PMC4631711

[B49] Kim J. Ross J. S. Kapczynski A. (2017). Pediatric exclusivity and regulatory authority: implications of Amgen v HHS. *JAMA* 319 21–22. 10.1001/jama.2017.16477 29117365

[B50] Knoppers B. M. Avard D. Sénécal K. Zawati M. H. (2014). Return of whole-genome sequencing results in paediatric research: a statement of the P3G international paediatrics platform. *Eur. J. Hum. Genet.* 22 3–5. 10.1038/ejhg.2013.176 23921532 PMC3865393

[B51] Knoppers B. M. Sénécal K. Boisjoli J. Borry P. Cornel M. C. Fernandez C. V. (2016). Recontacting pediatric research participants for consent when they reach the age of majority. *IRB: Ethics Hum. Res.* 38 1–9. 30088377

[B52] Knoppers B. M. Thorogood A. M. (2017). Ethics and big data in health. *Curr. Opin. Syst. Biol.* 4 53–57. 10.1016/j.coisb.2017.07.001

[B53] Knoppers B. M. Zawati M. H. Sénécal K. (2015). Return of genetic testing results in the era of whole-genome sequencing. *Nat. Rev. Genet.* 16 553–559. 10.1038/nrg3960 26239711

[B54] Lepola P. Needham A. Mendum J. Sallabank P. Neubauer D. de Wildt S. (2016). Informed consent for paediatric clinical trials in Europe. *Arch. Dis. Childhood* 101 1017–1025. 10.1136/archdischild-2015-310001 27226526 PMC5136704

[B55] Liberman D. B. Pham P. K. Nager A. L. (2014). Pediatric advance directives: parents’ knowledge, experience, and preferences. *Pediatrics* 134 e436–e443. 10.1542/peds.2013-3124 25002672

[B56] Lievens E. Milkaité I. (2017). *Age of Consent in the GDPR: Updated Mapping of Recent National Guidance and Proposals*. *Better Internet for Kids.* Available at: https://biblio.ugent.be/publication/8528973/file/8528974.pdf (accessed July 5, 2018).

[B57] Mäki-Petäjä-Leinonen A. (2016). “Protecting a person with dementia through restrictions of freedom? Notions of autonomy in the theory and practice of elder care,” in *Subjectivity, Citizenship and Belonging in Law: Identities and Intersections*, eds Griffiths A. Mustasaari S. Mäki-Petäjä-Leinonen A. (New York, NY: Routledge), 146–170.

[B58] Mills M. A. (1997). Narrative identity and dementia: a study of emotion and narrative in older people with dementia. *Ageing Soc.* 17 673–698. 10.1017/S0144686X97006673

[B59] Murphy K. Jordan F. Hunter A. Cooney A. Casey D. (2015). Articulating the strategies for maximising the inclusion of people with dementia in qualitative research studies. *Dementia* 14 800–824. 10.1177/1471301213512489 24403314

[B60] National Academies of Sciences, Engineering, and Medicine [NASEM] (2018a). *Open Science by Design: Realizing a Vision for 21st Century Research.* Washington, DC: National Academies Press. 10.17226/25116 30212065

[B61] National Academies of Sciences, Engineering, and Medicine [NASEM] (2018b). *Returning Individual Research Results to Participants: Guidance for a New Research Paradigm*, eds Botkin J. R. Mancher M. Busta E. R. Downey A. S. Washington, DC: National Academies Press. 10.17226/25094 30001048

[B62] NIH (2017). *NIH Policy and Guidelines on the Inclusion of Individuals Across the Lifespan as Participants in Research Involving Human Subjects.* Bethesda, MD: National Institutes of Health Available at: https://grants.nih.gov/grants/guide/notice-files/NOT-OD-18-116.html (accessed February 1, 2019).

[B63] Nishimura A. Carey J. Erwin P. J. Tilburt J. C. Murad M. H. McCormick J. B. (2013). Improving understanding in the research informed consent process: a systematic review of 54 interventions tested in randomized control trials. *BMC Med. Ethics* 14:28. 10.1186/1472-6939-14-28 23879694 PMC3733934

[B64] Nuffield Council on Bioethics (2009). *Dementia: Ethical Issues.* Available at: http://nuffieldbioethics.org/wp-content/uploads/2014/07/Dementia-report-Oct-09.pdf (accessed November 1, 2018).

[B65] Ontario Health Care Consent Act (2017). Available at: https://www.canlii.org/en/on/laws/stat/so-1996-c-2-sch-a/latest/so-1996-c-2-sch-a.html (accessed May 5, 2017).

[B66] Palmer B. W. Harmell A. L. (2016). Assessment of healthcare decision-making capacity. *Arch. Clin. Neuropsychol.* 31 530–540. 10.1093/arclin/acw051 27551024 PMC5007079

[B67] Palmer B. W. Harmell A. L. Pinto L. L. Dunn L. B. Kim S. Y. H. Golshan S. (2017). Determinants of capacity to consent to research on Alzheimer’s disease. *Clin. Gerontol.* 40 24–34. 10.1080/07317115.2016.1197352 28154452 PMC5279898

[B68] Palmer B. W. Savla G. N. (2007). The association of specific neuropsychological deficits with capacity to consent to research or treatment. *J. Int. Neuropsychol. Soc.* 13 1047–1059. 10.1017/S1355617707071299 17942022

[B69] Pediatric Trials Network Data Sharing (2018). Available at: https://www.pediatrictrials.org/data-sharing-opportunities/ (accessed November 8, 2018).

[B70] Proctor G. (2001). Listening to older women with dementia: relationships, voices and power. *Disabil. Soc.* 16 361–376. 10.1080/09687590120045932

[B71] Prusaczyk B. Cherney S. M. Carpenter C. R. DuBois J. M. (2017). Informed consent to research with cognitively impaired adults: transdisciplinary challenges and opportunities. *Clin. Gerontol.* 40 63–73. 10.1080/07317115.2016.1201714 28452628 PMC5911394

[B72] Rahimzadeh V. Schickhardt C. Knoppers B. M. Sénécal K. Vears D. F. Fernandez C. V. (2018). Key implications of data sharing in pediatric genomics. *JAMA Pediatr.* 172:476. 10.1001/jamapediatrics.2017.5500 29554172

[B73] Rahimzadeh V. Sénécal K. Kleiderman E. Knoppers B. M. (2017). *Minors and Incompetent Adults: A Tale of TWO populations.* Oxford: Oxford University Press 10.1093/oso/9780198786832.003.0019

[B74] Rigter T. Henneman L. Kristoffersson U. Hall A. Yntema H. G. Borry P. (2013). Reflecting on earlier experiences with unsolicited findings: points to consider for next-generation sequencing and informed consent in diagnostics. *Hum. Mutat.* 34 1322–1328. 10.1002/humu.22370 23784691 PMC4285964

[B75] Ross F. Donovan S. Brearley S. Victor C. Cottee M. Crowther P. (2005). Involving older people in research: methodological issues. *Health Soc. Care Commun.* 13 268–275. 10.1111/j.1365-2524.2005.00560.x 15819748

[B76] Shepherd V. (2016). Research involving adults lacking capacity to consent: the impact of research regulation on ‘evidence biased’ medicine. *BMC Med. Ethics* 17:55. 10.1186/s12910-016-0138-9 27609355 PMC5016956

[B77] Simpson A. R. (2015). Barriers and facilitators to the consent process in a study of complex genetic factors. *Am. J. Bioethics* 15 89–90. 10.1080/15265161.2015.1011013 25856621

[B78] Sweet L. Adamis D. Meagher D. J. Davis D. Currow D. C. Bush S. H. (2014). Ethical challenges and solutions regarding delirium studies in palliative care. *J. Pain Symptom Manage.* 48 1–17. 10.1016/j.jpainsymman.2013.07.017 24388124 PMC4082407

[B79] Taichman D. B. Sahni P. Pinborg A. Peiperl L. Laine C. James A. (2017). Data sharing statements for clinical trials: a requirement of the international committee of medical journal editors. *JAMA* 317 2491–2492. 10.1371/journal.pmed.1002315 28586895

[B80] Tait A. R. Voepel-Lewis T. Malviya S. (2007). Presenting research information to children: a tale of two methods. *Anesth. Anal.* 105 358. 10.1213/01.ane.0000270326.44507.11 17646490

[B81] Thorogood A. Dalpé G. McLauchlan D. Knoppers B. (2018a). Canadian consent and capacity regulation: undermining dementia research and human rights? *McGill J. Law Health* 12:67.

[B82] Thorogood A. Mäki-Petäjä-Leinonen A. Brodaty H. Dalpé G. Gastmans C. Gauthier S. (2018b). Consent recommendations for research and international data sharing involving persons with dementia. *Alzheimer’s Dementia* 14 1334–1343. 10.1016/j.jalz.2018.05.011 30293575

[B83] Thorogood A. Deschênes St-Pierre C. Knoppers B. M. (2017). Substitute consent to data sharing: a way forward for international dementia research? *J. Law Biosci.* 4 133–158. 10.1093/jlb/lsw063 28852560 PMC5570693

[B84] Thorogood A. Mäki-Petäjä-Leinonen A. Dalpé G. Gastmans C. Gauthier S. (2019). Openness, inclusion, and respect in dementia research. *Lancet Neurol.* 18 135–136. 10.1016/S1474-4422(18)30445-9 30663603

[B85] Tri-Council Policy Statement [TCPS] (2014). *Ethical Conduct for Research Involving Humans.* Available at: http://www.pre.ethics.gc.ca/pdf/eng/tcps2-2014/TCPS_2_FINAL_Web.pdf (accessed June 26, 2015).

[B86] UC SanDiego (2019). *Decision Making Capacity Guidelines. Human Research Protections Program (HRPP).* Available at: https://irb.ucsd.edu/Decisional-Capacity-Assessment.shtml (accessed January 17, 2019).

[B87] UK government (2005). *Mental Capacity Act 2005.* Available at: http://www.legislation.gov.uk/ukpga/2005/9/section/3 (accessed March 3, 2017).

[B88] UNESCO (2000). *Report on Confidentiality and Genetic Data.* Paris: United Nations Educational, Scientific and Cultural Organization (UNESCO) Available at: http://unesdoc.unesco.org/images/0013/001323/132334e.pdf (accessed December 8, 2017).

[B89] UNESCO (2003). *International Declaration on Human Genetic Data.* Available at: http://unesdoc.unesco.org/images/0013/001342/134217e.pdf (accessed August 27, 2013). 10.1163/157180904323042399 15285199

[B90] United Nations (1991). *United Nations Principles for Older Persons.* Available at: http://www.ohchr.org/Documents/ProfessionalInterest/olderpersons.pdf (accessed September 8, 2017).

[B91] United Nations (2014). *Committee on the Rights of Persons with Disabilities – General Comment No. 1.* Available at: https://documents-dds-ny.un.org/doc/UNDOC/GEN/G14/031/20/PDF/G1403120.pdf?OpenElement (accessed April 27, 2017).

[B92] United Nations Convention on the Rights of Persons with Disabilities [CRDP] (2006). Available at: http://www.un.org/disabilities/documents/convention/convoptprot-e.pdf (accessed March 2, 2017).

[B93] United Nations Treaty Collection (1989). *Convention on the Rights of the Child, United Nations, New York, United Nations, Treaty Series*, Vol. 1577 New York, NY: United Nations General Assembly, 3

[B94] United Nations Economic and Social Council (2004). *Resolution 2004/9 on Genetic Privacy and Non-discrimination.* Available at: http://www.un.org/en/ecosoc/docs/2004/resolution%202004-9.pdf (accessed March 13, 2017).

[B95] van El C. G. Cornel M. C. Borry P. Hastings R. J. Fellmann F. Hodgson S. V. (2013). Whole-genome sequencing in health care: recommendations of the European society of human genetics. *Eur. J. Hum. Genet.* 21 S1–S5. 10.1038/ejhg.2013.46 23819146 PMC3660957

[B96] Waldron J. (2017). *One Another’s Equals: The Basis of Human Equality*, 1st Edn. Cambridge, MA: Belknap Press 10.4159/9780674978867

[B97] Warner J. Nomani E. (2008). Giving consent in dementia research. *Lancet* 372 183–185. 10.1016/S0140-6736(08)61049-1 18640439

[B98] Wendler D. S. (2006). Assent in paediatric research: theoretical and practical considerations. *J. Med. Ethics* 32 229–234. 10.1136/jme.2004.011114 16574878 PMC2588342

[B99] Wilfond B. S. Diekema D. S. (2012). Engaging children in genomics research: decoding the meaning of assent in research. *Genet. Med.* 14 437–443. 10.1038/gim.2012.9 22323071 PMC6317361

[B100] Wolf S. M. Scholtes E. Koenig B. A. Petersen G. M. Berry S. A. Beskow L. M. (2018). Pragmatic tools for sharing genomic research results with the relatives of living and deceased research participants. *J. Law Med. Ethics* 46 87–109. 10.1177/1073110518766024 30008546 PMC6040667

[B101] World Medical Association [WMA] (2013). *Declaration of Helsinki – Ethical Principles for Medical Research Involving Human Subjects.* Available at: https://www.wma.net/policies-post/wma-declaration-of-helsinki-ethical-principles-for-medical-research-involving-human-subjects/ (accessed October 19, 2017).

[B102] Zawati M. H. Parry D. Thorogood A. Nguyen M. T. Boycott K. M. Rosenblatt D. (2014). Reporting results from whole-genome and whole-exome sequencing in clinical practice: a proposal for Canada? *J. Med. Genet.* 51 68–70. 10.1136/jmedgenet-2013-101934 24078715

